# An integrated strategy for target SSR genotyping with toleration of nucleotide variations in the SSRs and flanking regions

**DOI:** 10.1186/s12859-021-04351-w

**Published:** 2021-09-08

**Authors:** Yongxue Huo, Yikun Zhao, Liwen Xu, Hongmei Yi, Yunlong Zhang, Xianqing Jia, Han Zhao, Jiuran Zhao, Fengge Wang

**Affiliations:** 1 Maize Research Center, Beijing Academy of Agricultural and Forest Sciences (BAAFS)/Beijing Key Laboratory of Maize DNA Fingerprinting and Molecular Breeding, Beijing, 100097 China; 2 Provincial Key Laboratory of Agrobiology, Institute of Crop Germplasm and Biotechnology, Jiangsu Academy of Agricultural Sciences, Nanjing, 210014 Jiangsu China

**Keywords:** SSR-GBS, Algorithm, Microsatellite, Sequence-based microsatellite genotyping, Genetic analysis

## Abstract

**Background:**

With the broad application of high-throughput sequencing and its reduced cost, simple sequence repeat (SSR) genotyping by sequencing (SSR-GBS) has been widely used for interpreting genetic data across different fields, including population genetic diversity and structure analysis, the construction of genetic maps, and the investigation of intraspecies relationships. The development of accurate and efficient typing strategies for SSR-GBS is urgently needed and several tools have been published. However, to date, no suitable accurate genotyping method can tolerate single nucleotide variations (SNVs) in SSRs and flanking regions. These SNVs may be caused by PCR and sequencing errors or SNPs among varieties, and they directly affect sequence alignment and genotyping accuracy.

**Results:**

Here, we report a new integrated strategy named the accurate microsatellite genotyping tool based on targeted sequencing (AMGT-TS) and provide a user-friendly web-based platform and command-line version of AMGT-TS. To handle SNVs in the SSRs or flanking regions, we developed a broad matching algorithm (BMA) that can quickly and accurately achieve SSR typing for ultradeep coverage and high-throughput analysis of loci with SNVs compatibility and grouping of typed reads for further in-depth information mining. To evaluate this tool, we tested 21 randomly sampled loci in eight maize varieties, accompanied by experimental validation on actual and simulated sequencing data. Our evaluation showed that, compared to other tools, AMGT-TS presented extremely accurate typing results with single base resolution for both homozygous and heterozygous samples.

**Conclusion:**

This integrated strategy can achieve accurate SSR genotyping based on targeted sequencing, and it can tolerate single nucleotide variations in the SSRs and flanking regions. This method can be readily applied to divergent sequencing platforms and species and has excellent application prospects in genetic and population biology research. The web-based platform and command-line version of AMGT-TS are available at https://amgt-ts.plantdna.site:8445 and https://github.com/plantdna/amgt-ts, respectively.

**Supplementary Information:**

The online version contains supplementary material available at 10.1186/s12859-021-04351-w.

## Background

Simple sequence repeats (SSRs), also named microsatellites or short tandem repeats (STRs), can be widely found in eukaryotic genomes [1]. The sequences that flank an SSR may be sufficiently conserved to allow specific amplification primers to be designed; thus, SSRs can be detected through conventional PCR amplification and typed based on the amplification products. The majority of SSRs are noncoding and thus can affect the expression, splicing, protein sequence, and genome structure of genes [2, 3]. SSRs makers are commonly used in genome-related studies [4, 5]. SSR genotyping has also become an extensive application in different fields and has been used for population genetic diversity and structure analysis, the construction of genetic maps, and the investigation of intraspecies relationships [6–8].

**Fig. 1:**
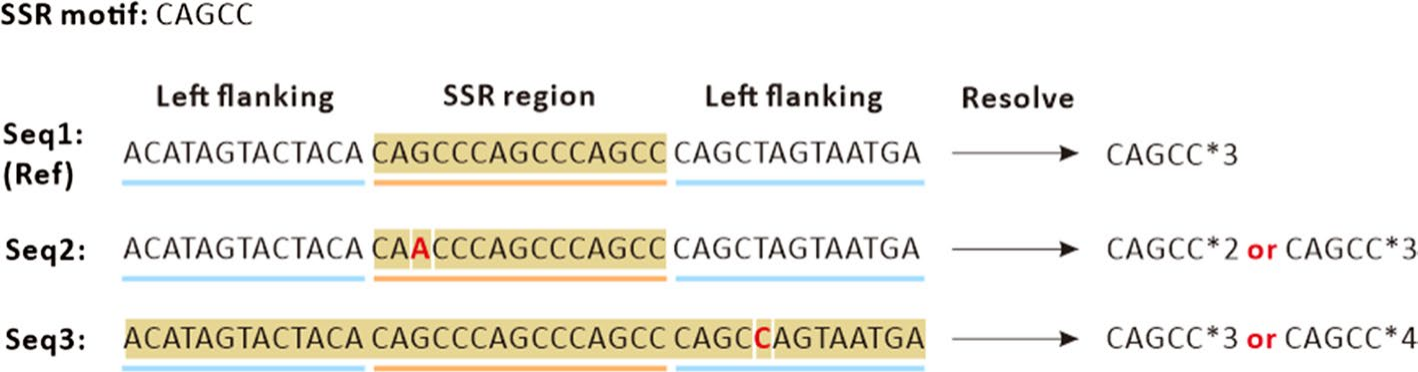
Schematic diagram of error-prone SSR typing caused by variations in SSR or flanking regions. Take a site with CAGCC SSR motif as an example, for Seq1 (from reference genome), it’s clear that its SSR region is with three times repeats of CAGCC; for Seq2 with a G- > A variation in SSR region, the regular exact matching algorithm will type it as two repeats of CAGCC, while fault-tolerant algorithm could recognize it as three repeats; for Seq3 with a T- > C variation in right flanking region, flanking boundary-based algorithm will treat it as three repeats of CAGCC, however, the regular exact matching algorithm will recognize it as four times of repeat. When comparing different samples, especially different varieties, this discordance of SSR typing will cause misunderstanding of genetic information

All applications of SSRs are based on accurate SSR genotyping methods, and less accuracy may have serious consequences [9, 10]. Moreover, the construction and application of DNA databases also require the accurate SSR genotyping of samples [11, 12]. Factors influencing accurate SSR genotyping include the following: 1) The slippage of polymerase is inherent to in vitro SSR polymerase PCR amplification, which leads to incorrect SSR alleles and makes it challenging to genotype SSRs accurately; and 2) the occurrence of variations in the SSR or flanking region will directly affect the genotyping results (Fig. 1) [13, 14]. These problems accompanied the SSR genotyping technology development. The technology has experienced the initial gel electrophoresis, capillary electrophoresis, the first- and second-generation sequencing, and the high-throughput amplicon sequencing stage. At present, amplicon sequencing technology is widely used in genetic disease screening and gene diagnosis, as well as in other research [15, 16]. However, there is still no suitably accurate SSR genotyping method that can tolerate nucleotide variations in SSRs and flanking regions which may affect the sequence alignment and genotyping accuracy.

Here, we developed a new open-source microsatellite genotyping strategy that includes an accurate microsatellite genotyping tool based on targeted sequencing (AMGT-TS) and a user-friendly web-based version. AMGT-TS can quickly perform precise SSR genotyping with ultradeep coverage and high locus throughput, and it includes a broad matching algorithm (BMA) that can handle situations with nucleotide variations in the SSR and flanking regions. We also performed a comprehensive assessment of AMGT-TS using internal laboratory testing and simulated data testing. The results showed that AMGT-TS could achieve nearly 100% typing accuracy. Although AMGT-TS developed on plants, which is the focus of our current work, the new method is generic and can be used as a new tool for many biological fields. All completed codes, sample data, and documentation have been submitted to GitHub.

### Implementation

#### AMGT-TS tool design

The process of AMGT-TS has three main steps to obtain accurate genotyping information (Fig. 1). For each sample: first, reads are mapped to their *bona fide* loci according to the reference sequences; then, the SSR regions are determined by the loci’s flanking information; and finally, AMGT-TS obtains accurate SSR genotyping results based on the dissection of read information, such as read number and primary SSR typing.

In detail, after obtaining raw sequencing data (usually in FASTQ format), we use FASTX (http://hannonlab.cshl.edu/fastx_toolkit/) to remove low-quality data. Then, we perform the "Alignment to loci" processing step with bwa-mem [17], based on the read information for the loci in the reference sequence file. After this step, reads will be grouped to a locus. Next, Picard (https://broadinstitute.github.io/picard/) is used to group reads in the same locus together. At the same time, SAMtools [18] is used to index the data to improve the efficiency of subsequent processing. Next, we use SAMtools to “Split by direction,” separating the forward and reverse data. Then, we use SEQTK (https://github.com/lh3/seqtk) to “Adjust direction,” which flips reverse sequences into forward sequences. After that, we use the BLAST tool [19] to perform the “Find SSR region” operation according to the 20-bp sequences of the left and right flanking sequences of SSR regions in the reference sequence to obtain the SSR region of each read. Finally, we use Python scripts to “Find SSR typing” in the SSR region to obtain SSR typing information.

**Fig. 2:**
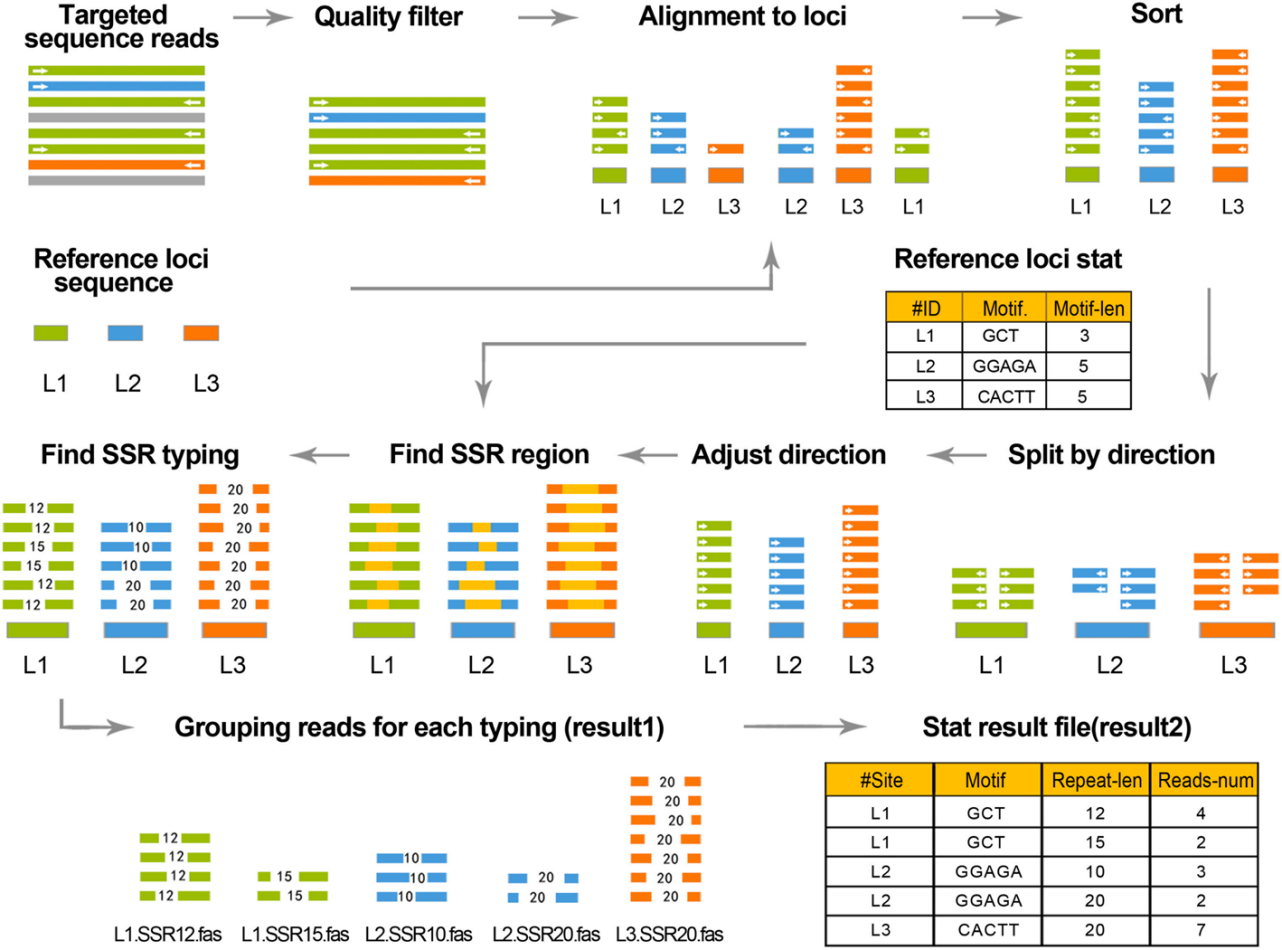
The processing flow of AMGT-TS. Green bar: reads of Locus 1 (L1), blue bar: reads of Locus 2 (L2), orange bar: reads of Locus 3 (L3). Gray bars indicate low-quality reads. The solid arrow represents step-by-step operations in the process. The dotted arrows represent the data information referenced by the corresponding step. The small white arrows within the color bars pointing to the right represent forward sequences and those pointing to the left represent reverse sequences

**Fig. 3:**
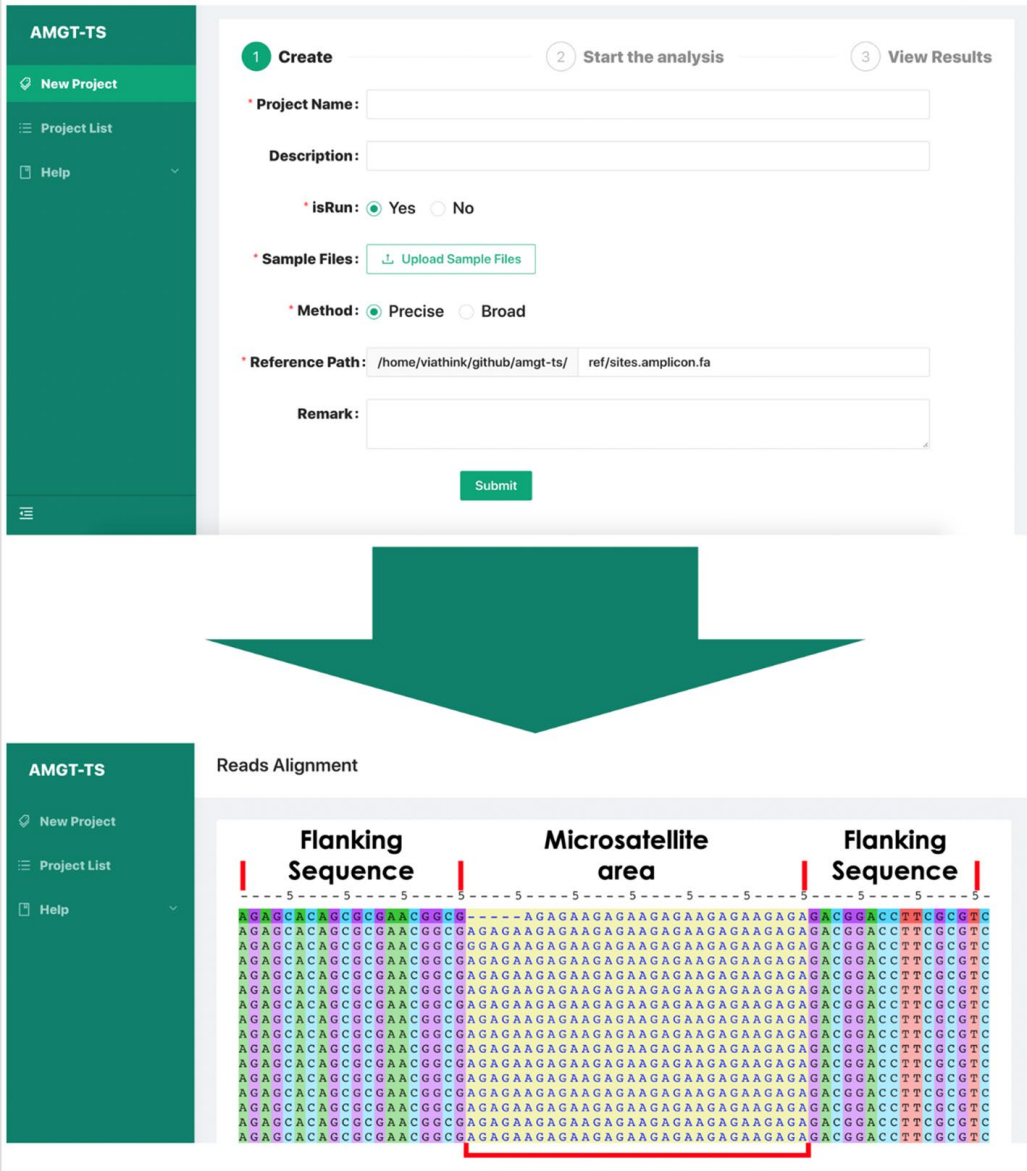
Read alignment of locus s4121 for the motif AGAGA of B73 repeated six times (SSR region of 30 bp; yellow background)

Here, as an example of the AMGT-TS processing workflows, two actual experimental datasets are provided for result 1 and 2 in Fig. 2. For result 1, an SSR genotyping result of AGAGA*6 for locus s4121 in B73 (a model variety of maize) is shown (Fig. 3). We used the AMGT-TS web platform (https://amgt-ts.plantdna.site:8445/) to generate the alignment figure for the reads. The platform grouped the read files to align the heap sequences in Fig. 3, which shows the results of the classification alignment. Each line is one read, and the yellow background region is the SSR region being typed. The genotype is exactly six repeats of 5 bases. For result 2, the typing result of locus s17883 is shown in Additional file 1: Table S1. An SSR length of 12 (ATA*4) was found for 98.20% of the reads, so we can obtain the result ATA (4,4) (maize is a diploid plant, so each locus has two alleles). In addition, we obtained the SSR typing result of AGG (4,4) for locus s691405. Finally, the genotype of the third locus (s838417) was a homozygous type CTC (5,5), which is a 15 bp long repeat, and the corresponding reads accounted for 98.70% of the total reads. Overall, the typing strategy of AMGT-TS is clear and satisfactory.

### Evaluation of typing error

The typing error can be measured in two ways. One is the false-positive rate of SSR typing and the other is the rate of the error reads found for the correct typing result. Equation (1) can be obtained, where *j* represents the index of the locus, *k* represents the typing index, and *R*_*amgt*_*(j, k)* represents the reads of the *k*-th typing of the *j*-th locus from AMGT-TS.

1 $${\text{Sum }}\;{\text{of}}\;{\text{R}}_{{{\text{amgt}}}} = \mathop \sum \limits_{{\text{j}}} \mathop \sum \limits_{{\text{k}}} {\text{R}}_{{{\text{amgt}}}} \left( {{\text{j}},{\text{k}}} \right)$$

*S*_*ra*_ represents the sum of reads from artificial data and *E*_*r*_ represents the error of the reads. Equation (2) can be obtained as follows:

2 $$E_{r} = \frac{{{\text{S}}_{{{\text{ra}}}} { } - { }\mathop \sum \nolimits_{{\text{j}}} \mathop \sum \nolimits_{{\text{k}}} {\text{R}}_{{{\text{amgt}}}} \left( {{\text{j}},{\text{k}}} \right)}}{{{\text{S}}_{{{\text{ra}}}} }}$$

In the same way, *E*_*t*_ represents typing error, *T*_*a*_ represents the sum of typing of artificial data and *T*_*amgt*_ represents the correct typing result count from AMGT-TS. Equation (3) can be obtained as follows:

3 $$E_{t} = \frac{{{\text{T}}_{{\text{a}}} { } - { }\mathop \sum \nolimits_{{\text{j}}} {\text{T}}_{{{\text{amgt}}}} \left( {\text{j}} \right)}}{{{\text{T}}_{{\text{a}}} }}$$

### Precise and broad matching algorithms

**Fig. 4:**
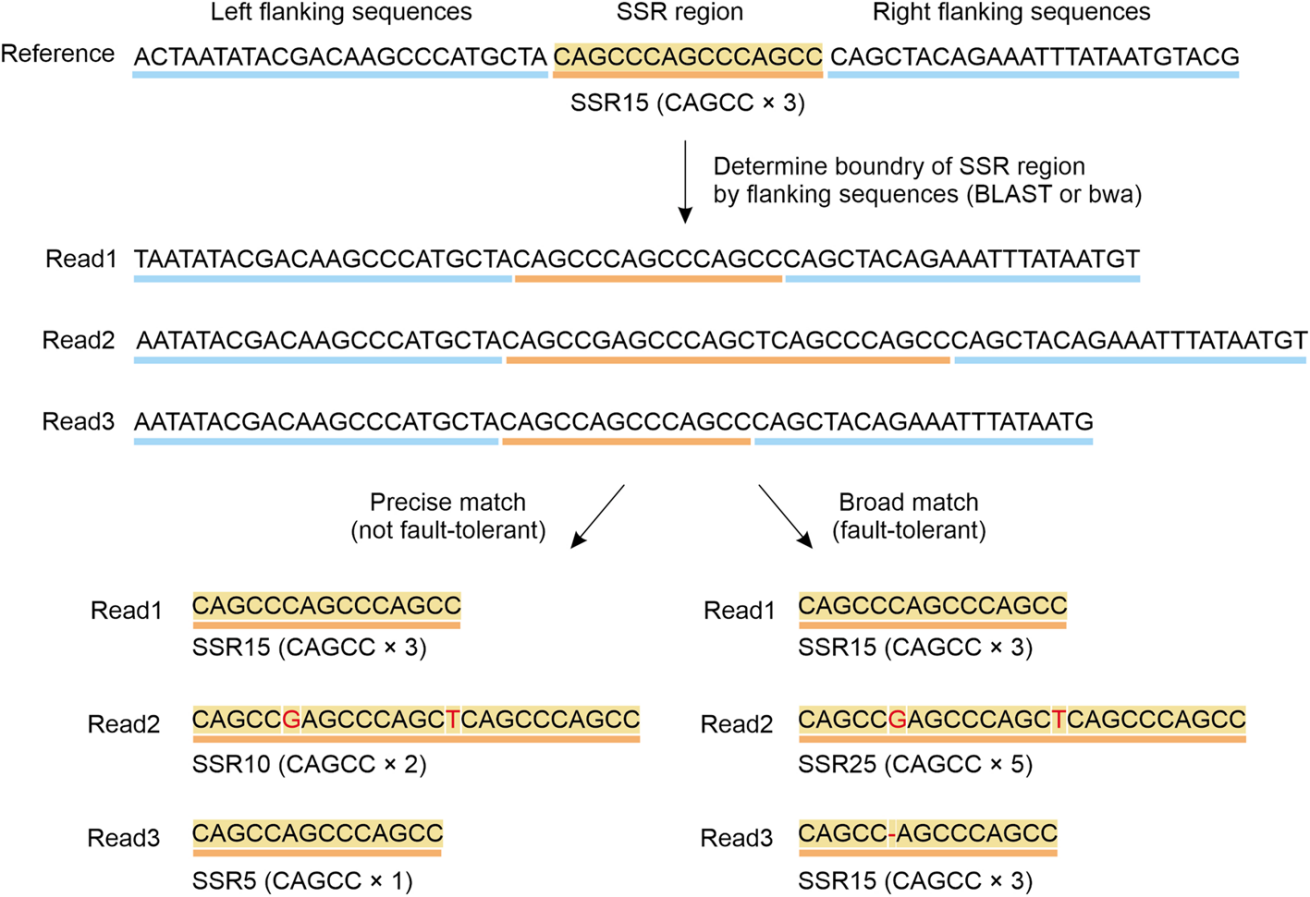
The different approaches of precise and broad matching strategies. The arrow to the left is pointing the result of the precise method, and the right to the broad method. We can see genotyping of Read1 and Read3 is the same, but not with Read2. For there are variants in the SSR region, the precise method can only identify 2 motif repeats, while the broad method can identify 5 repeats

To archive both the high accuracy of SSR typing and tolerance of the variations in SSRs and flanking regions, we respectively developed two different algorithms, precise and broad matching strategies (Fig. 4). The analytical strategy of precise matching is divided into three steps. The first step is "grouping." For multilocus amplicon sequencing data, sequencing reads are first assigned to the corresponding loci according to the reference sequences. AMGT-TS uses bwa-mem to implement data mapping. The second step is "SSR boundary determination". After extraction of sequences for each locus, AMGT-TS uses flanking sequences of the SSR region of each locus in the reference sequences to determine the boundaries of the left and right flanking sequences, which indirectly determines the boundaries of the SSR region and further extracts the sequence of the SSR region by calling BLAST. The third step is "SSR genotyping." After the SSR sequence has been determined, the repetition number of SSRs is determined by using the precise match method of repeated sequences, and the SSR repetition length is used to name the SSR genotype. For example, the motif of a certain SSR was ATC, with a repetition number of three times, so the SSR was named SSR9.

The broad matching algorithm (BMA) has the same first step as the precise matching algorithm. However, in the second step, BMA directly processes the information of BAM files and uses the Concise Idiosyncratic Gapped Alignment Report (CIGAR) information for each read to mask the classification information, which makes it compatible with the variation within a certain error range and results in better fault-tolerant classification information. As shown in Fig. 4, the SSR motif represents the repeating unit in the SSR region. The SSR region represents the region where the SSR sequence is located. For example, when a sequence of an SSR is AGCAGCAGC, the SSR motif is AGC, and the SSR region is AGCAGCAGC. The precise match identifies only contiguous motifs, so in read 2, only the last 10 bp is identified as two replicates. Read 3 has only one motif repeat. For the broad match, the results identified were identical to those for the precise match, except for read 1, which is a perfectly repeated sequence; different results were obtained for the other two reads. For read 2, when the two red bases are considered to be two SNPs, 5 repeats of the motif are obtained. For read 3, when the region is considered to be an InDel, is the motif is considered to contain 3 repeats.

### Simulation test of AMGT-TS

To better simulate different situations, each read was divided into five parts (Additional file 1: Figure S1). Five different categories of reads were considered in our simulation, named Classes A to E. The detailed method of generating these data was as follows:

- Class A: We created the SSR_Region according to 3 repeats of the motif of s17883. We then added 35 bp from the left and right flanking sequences of the SSR_region of the reference sequence as Flank_L and Flank_R, respectively. Finally, we added one SNP on the left flank and one SNP on the right flank. There were 2000 artificial reads for this dataset.
- Class B: We created the SSR_Region according to 5 repeats of the motif (AGCT) and 6 repeats of the motif of s423645. We then added 35 bp from the left and right flanking sequences of the SSR_region of the reference sequence as Flank_L and Flank_R, respectively. There were 1000 artificial reads for this dataset.
- Class C: We created the SSR_Region according to 4 repeats of the motif (CGCAT) and 3 repeats of the motif (CGCAT) + CACAT + 2 repeats of motif s566749. We then added 35 bp from the left and right flanking sequences of the SSR_region of the reference sequence as Flank_L and Flank_R, respectively. There were 2000 artificial reads for this dataset.
- Class D: In the SSR_Region of this type, Flank_L and Flank_R are also random bases. With the addition of Random_L and Random_R, the total length was randomly extended to 180 ~ 220 bp. There were 1000 artificial reads for this dataset.
- Class E: The rule is the same as for Class A, but There were 2000 artificial reads for this dataset. Random_L and Random_R are random bases and the total length was randomly extended to 180 ~ 220 bp.

Classes A to C combined contained a total of 8000 reads. Random_L and Random_R regions were random bases, and the total length was randomly extended to 180 ~ 220 bp. Classes A to D combined contained a total of 9000 reads. The quality information from Class A to Class D was marked as the highest. The quality information of Class E was marked as the lowest. For the read numbering rule, numbering is divided into three segments. The first segment is fixed: @BMSTC (Beijing Maize Seed Testing Center), representing the artificial sequence. The second segment is category information, using 1, 2, 3, 4, and 5 to represent A, B, C, D, and E, respectively. The third is the ordinal number, starting from 1 in each category and ending with the maximum number of entries in the current category. After 10,000 reads were created, they were randomly distributed into FASTQ files.

### Software and package dependencies

AMGT-TS was verified on Ubuntu Server 14.04.4 LTS and 18.04.2 LTS. AMGT-TS relies on various tools including Bamtools (v2.5.0) [20], BLAST tool suite (v2.6.0 +) [19], BWA (v0.7.17-r1188) [17], fastx_toolkit (v0.0.13), Picard (v2.15.0), SAMtools (v1.3.1) [18], and SEQTK (v1.2). The Java version used in AMGT-TS is OpenJDK1.7. The Python version is 2.7 + . Pandas are required in Python and can be installed using PIP.

### AMGT-TS implementation details

AMGT-TS runs on Linux, and Ubuntu 18.04 has been tested. After downloading the code from GitHub, the user needs to install the dependent components as described in the README.md file. The ENV_FILE variable in the launch.sh specifies the location of the configuration file. In the configuration file, the user must configure the corresponding component location. The targeted sequencing sample files are placed in the “working/00_fastq” directory. Under the directory “REF_DIR” of the configuration file is the reference sequence file information for each locus. Once this information is configured, the user can execute the launch.sh file to run the tool. Before running the program, the user can specify the different algorithms: precise or broad. When the tool is finished running, a log file will be generated. In the “working/04_reads” directory, the locus typing information of the whole sample is present. In the directory of each locus is the typing information of the current loci and reads corresponding to each type. For each subheap read file, the user can use the Reads Alignment tool for a graphical presentation.

## Results

### Overview

Currently, no suitable genotyping method can achieve tolerance of single nucleotide variations (SNVs) in the SSRs and flanking regions, which may be caused by PCR and sequencing errors or SNPs among varieties and can directly affect the sequence alignment and genotyping accuracy. As shown in Fig. 1, taking a site with a CAGCC SSR motif as an example, for Seq1 (from the reference genome), its SSR region has three repeats of CAGCC; for Seq2 with a G- > A variation in the SSR region, the regular exact matching algorithm will type it as two repeats of CAGCC, while the fault-tolerant algorithm can recognize it as three repeats; and for Seq3 with a T- > C variation in right flanking region, the flanking boundary-based algorithm will treat it as three repeats of CAGCC, however, the regular exact matching algorithm will recognize it as four repeats. When comparing different samples, especially different varieties, this discordance in SSR typing will cause a misclassification of the genetic information.

To address this issue, in this study, we developed a broad matching algorithm (BMA) that can quickly and accurately achieve SSR typing for ultradeep coverage and high-throughput loci with SNV compatibility and grouping of the typed reads for further in-depth information mining. We also designed the AMGT-TS tool incorporating the BMA for targeted microsatellite genotyping. Below, we tested the AMGT-TS tool using both experimental data and simulated data. We also compared AMGT-TS with other SSR-typing tools, as well as the popular commercial SSR-typing software, NextGENe.

### Experimental evaluation

**Fig. 5:**
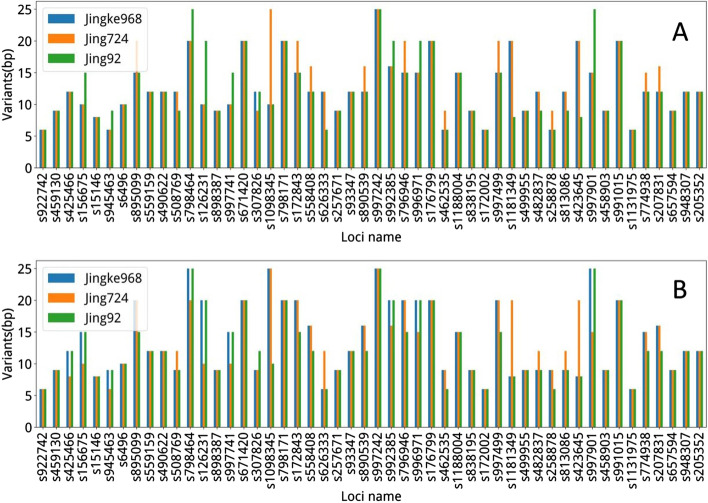
Allele variants for each locus of three example samples detected by AMGT-TS. To evaluate the AMGT-TS tool, an example for analyzing the three samples with a genetic relationship is given and a total of 50 loci is selected to verify the genetic relationship. The three samples are Jingke968 and its parents Jing724 (female parent) and Jing 92 (male parent). To visually observe the genetic compatibility, panels (**A**) and (**B**) refer to the first and second allele results of the 50 loci, respectively. In the figure, the abscissa shows the 50 loci; the longitudinal coordinates are the length of the genotyping fragment (bp) of each sample. The 50 loci are 100% following the genetic relationship, indicating that the AMGT-TS analysis results are accurate

We used three genetically related samples to map the genotyping information of 50 loci (Fig. 5 and Additional file 1: Table S2). The typing results of the offspring samples were 100% found in the two parents, indicating that the typing outcomes of AMGT-TS are precise and that AMGT-TS is potentially useful for genetic analysis. Furthermore, we used AMGT-TS to analyze targeted sequencing data of 8 samples and 21 randomly sampled loci and compared the results with resequencing results (Additional file 1: Figure S2, Table S3 and S4). We compared loci that produced valid data at the same locus in both experiments. If any experiment did not produce a result at a certain locus, then the locus was not included in the comparison. In Additional file 1: Fig. S2, the minimum number of loci for comparison in the sample was 11, and the maximum number was 18. The results of all compared loci were 100% consistent.

### Simulated data test

To further verify the precision of the AMGT-TS results, we used a manual method to create the original data of simulated targeted sequencing with 10,000 reads. The average read length of these data was around 200 bp, based on the information of three example loci (s499955, s423645, and s996971) from B73 (details in Implementation, artificial read composition design in Additional file 1: Figure S1).

For the simulated data, the results of AMGT-TS analysis are shown in Additional file 1: Table S5. Using the above calculation for the error rate evaluation, we obtained *E*_*r*_ = 0 and *E*_*t*_ = 0; in other words, for the typing results of the simulated targeted sequencing data, the accuracy of reads and SSR typing was 100%. As shown in Additional file 1: Table S5, 1000 low-quality points were filtered correctly, whereas 1000 random reads were not recognized. In addition, the precise matching algorithm did not deal with SNPs in the SSR region and identified these SRRs only as three repeats of the motif. However, the broad matching algorithm could tolerate these SNPs and identified these SSRs as 6 repeats of the motif, which are shown in Additional file 1: Figure S3A. Then Additional file 1: Figure S3B shows the situation with SNPs in the flanking region. The broad matching algorithm has robust fault tolerance.

### Comparison with other SSR-typing tools

To determine the detection accuracy of AMGT-TS, we made an integrated comparison with other published SSR typing tools, SSRseq [21], MicNeSs [22] and CHIIMP [23], with different simulated datasets (Table 1). To provide a more rational comparison, we carried out simulations based on three *bona fide* loci from the Maize B73 V3 reference genome (Additional file 1: Table S6); these three loci have different motif lengths (from 3 to 5 bp) and the different polymorphism information contents (PICs). For each locus, we simulated four situations (Additional file 1: Table S7): no variant in the SSR or flanking region (Dataset A, as a control), one SNP site in the SSR region (Dataset B), one SNP site in the flanking region (Dataset C) and a 2 bp deletion in the flanking region (Dataset D). There were 10,000 reads for each locus in each dataset. After simulation and SSR-typing by each tool (Table 1), we found that SSRseq has good performance on Datasets A to C, while it is poor for SSR tying of the locus with flanking variants. MicNeSs was designed to screen perfect SSR sites and has poor performance for SSR typing of the long motif (> 3 bp). CHIIMP has poor performance for SSR tying of the SSR region with SNPs. Among these four tools, only AMGT-TS can deal with all four situations; specifically, this tool has excellent performance for SSR tying of the loci with variants in flanking or SSR regions.

**Table 1:** Comparison of AMGT-TS with other SSR-typing tools

Tool	Dataset	Locus1		Locus2		Locus3		OS/development language	Note
		Correct/total	Accuracy (%)	Correct/total	Accuracy (%)	Correct/total	Accuracy (%)		
AMGT-TS (this study)	A	10 k/10 k	100	10 k/10 k	100	10 k/10 k	100	Linux/Python	High-throughput; good performance for SSR-tying of the locus with variants in flanking and SSR regions
	B	10 k/10 k	100	10 k/10 k	100	10 k/10 k	100		
	C	10 k/10 k	100	10 k/10 k	100	10 k/10 k	100		
	D	10 k/10 k	100	10 k/10 k	100	10 k/10 k	100		
SSRseq [21]	A	10 k/10 k	100	10 k/10 k	100	10 k/10 k	100	Linux/Python	High-throughput; poor performance for SSR-tying of the locus with flanking variants
	B	10 k/10 k	100	10 k/10 k	100	10 k/10 k	100		
	C	10 k/10 k	100	10 k/10 k	100	10 k/10 k	100		
	D	0/10 k	0	0/10 k	0	0/10 k	0		
MicNeSs [22]	A	10 k/10 k	100	0/10 k	0	0/10 k	0	Linux/Python	Reference-free and motif information-free; Only one locus per process; poor performance for SSR-tying of the long motif (> 3 bp)
	B	10 k/10 k	100	0/10 k	0	0/10 k	0		
	C	10 k/10 k	100	0/10 k	0	0/10 k	0		
	D	10 k/10 k	100	0/10 k	0	0/10 k	0		
CHIIMP [23]	A	10 k/10 k	100	10 k/10 k	100	10 k/10 k	100	Linux, Windows/R	Only one locus per process; poor performance for SSR-tying of SSR region with SNP variants
	B	0/10 k	0	0/10 k	0	0/10 k	0		
	C	10 k/10 k	100	10 k/10 k	100	10 k/10 k	100		
	D	10 k/10 k	100	10 k/10 k	100	10 k/10 k	100		

Dataset A: No variants in SSR region; Dataset B: One SNP in SSR region; Dataset C: One SNP in flanking; Dataset D: 2-bp deletion in flanking. Details of simulation information are listed in Additional file 1: Table S6 and S7 and simulation data was uploaded to https://amgt-ts.plantdna.cn/data/

We also made a comparison with a popular commercial SSR typing software, NextGENe (https://softgenetics.com/NextGENe.php), using three sets of Ion Torrent sequencing data (Additional file 1: Figure S4 and Additional file 2: Table S8-S13), including data from Jingke968, a hybrid from two different maize varieties; Jing724, a selfing variety; and the genome-sequenced variety B73. In a total of 484 evaluated SSR loci, more than 96% of the alleles detected by NextGENe were also detected by AMGT-TS. In contrast, more than 100 alleles were detected exclusively by AMGT-TS (Additional file 1: Figure S4). After manual validation, we confirmed that the missing alleles from the NextGENe results are caused by a short flank size: NextGENe cannot handle reads with only 5–330 bp on the left or right flank, while AMGT-TS can. Overall, these results show that AMGT-TS is accurate and highly capable of SSR variant genotyping detection.

## Discussion

The development of multiplex PCR technologies has made it possible to amplify multiple target sites at once. Moreover, the development of amplicon sequencing technology has made large-scale high-throughput SSR typing possible. At present, amplicon sequencing technology is widely used in genetic disease screening and gene diagnosis, as well as plant breeding [15, 16]. Our study breaks through the limitations of traditional typing methods and achieves large-scale typing of SSR at the single-base level; this method is fast, accurate, and low-cost, and can be widely applied in genetic diversity studies, highly precise gene localization, and molecular-assisted selection of new varieties [21]. Here, we propose a tool for developing new SSR-seq approaches and we demonstrated its efficiency for a range of species with different levels of genomic resource availability. The most important feature is that this tool provides strategies to optimize locus selection and primer design. This tool can be used for locus selection and merit selection. AMGT-TS can analyze three error-prone and complicated cases, including cases with too many dominant SSR types of certain loci, an extremely low ratio of reads of the dominant SSR types and too much variation within the SSR region. Then, researchers can treat these loci as low-quality loci based on the information provided by AMGT-TS. By filtering the above three types of information, high-quality SSR sites can be obtained, which is important for accurate typing [9, 10]. Since genotyping data consist of simple nucleotide character strings that do not need to be encoded or encapsulated in special data types, it is easier to use existing bioinformatics tools to perform pipelining, resulting in easier for data sharing between different laboratories and storage in different databases for different applications.

AMGT-TS can use the precise matching algorithm to accurately obtain SSR classification, based on the premise that there is no change in the SSR region. However, polymorphisms in the repeat motif are hard to determine and will affect the accuracy of SSR detection. When there are variations in the SSR region or base changes due to experiments, AMGT-TS can use the broad matching algorithm to account for the variation in the SSR region. The broad strategy used in AMGT-TS differs from the methods implemented in other SSR genotyping software, such as MicNeSs [22] which can also identify the SSR genotypes based on sequencing data while accounting for up to one substitution within the SSR regions. In addition, AmpSeq-SSR is a microsatellite genotyping tool with similar functions as AMGT-TS [24]. When AmpSeq-SSR encounters motif repeats with base variation in one of the intermediate repeats, the result is an identification errors and complete motif repeats are lost, thus directly affecting genotyping results. For AMGT-TS, resource data can be either FASTA or FASTQ files, and especially for FATSQ files, a quality-based filtering process could not only increase the accuracy of the results, but also reduce the analysis time. The data processed by AmpSeq-SSR are only in FASTA format, which contains no quality information, so the above optimization cannot be performed.

Usually, for ultradeep sequencing, the prominent peak(s) will be considered the *bona fide* SSR genotype(s). The remaining genotypes tend to be caused by amplification stutter or sequencing error. Take two loci as examples, as shown in the Additional file 1: Figure S5. Jing724 and Jingke968 are a selfing maize variety and a hybrid from two different maize varieties, respectively. Thus, loci in Jing724 and Jingke968 are expected to have one genotype and two genotypes, respectively. As found here, the s994429 locus in Jing724 and the s677195 locus in Jingke968 have one peak (TCAT*3) and two peaks (AAG*4 and AAG*6) detected by AMGT-TS, respectively. These results indicate that AMGT-TS has an excellent ability to accommodate amplification stutter or sequencing error.

Previous tools based on targeted sequencing can only identify consecutive SSR motifs. They cannot deal with cases where there is variation in the SSR region (possibly due to an experimentally introduced error) [22, 24]. In contrast, AMGT-TS can obtain consecutive SSR motif sequences and deal with cases containing variations in the SSR region, to allow us to have a clear, intuitive and comprehensive understanding of the actual situation of SSR genotyping. AMGT-TS is a powerful and robust tool for applications that require precise knowledge of SSR genotyping, such as diagnosing diseases. AMGT-TS has the robustness of classification recognition so that even when there are a few errors in the data, complete repetitive information is not lost. AMGT-TS analyzes the CIGAR information of the BAM file to carry out processing compatible with the variation in SSR regions. Furthermore, the different results produced by these two algorithms in AMGT-TS make a significant difference in the classification of plant varieties and disease detection. Therefore, different algorithms can be considered for various biological fields.

## Conclusion

In conclusion, the BMA and AMGT-TS tools provide an integrated strategy for accurate microsatellite typing for ultradeep coverage and high-throughput analysis of loci with SNV compatibility and grouping the typed reads for further in-depth information mining. With the broader application of next-generation sequencing techniques and the current application of AMGT-TS to divergent sequencing platforms and species, we expect that AMGT-TS will have excellent application prospects in genetic and population biology research in the future.

## Supplementary Information

**Additional file 1 **: **Figure S1**. Artificial read composition design; **Figure S2**. Comparison of targeted sequencing results and resequencing results (8 samples/21 loci). This figure shows a comparison of targeted sequencing results analyzed by AMGT-TS and resequencing results (8 samples/21 loci). The abscissa is the name of each sample. The orange ordinate represents the number of loci that were compared. The green ordinate represents the same number of compared loci. Loci with missing or incomplete data were not compared. In the figure above, refer to Table S1 for the corresponding resequencing data. For the corresponding data of targeted sequencing results analyzed by AMGT-TS, please refer to Table S2. For the loci information, please refer to Table S5; **Figure S3**. The situation with SNP in the SSR and flanking regions; **Figure S4**. Comparison of SSR genotyping results between AMGT-TS and NextGENe; **Figure S5**. SSR typing results of two representative loci by AMGT-TS; **Table S1**. Genotyping information for three example loci; **Table S2**. This table shows the results of genotyping of 50 loci from Figure 3; **Table S3**. Data of Figure S2 - Resequencing data of 8 samples; **Table S4**. Data of Figure S2 - Targeted sequencing results analyzed by AMGT-TS; **Table S5**. Analysis results of simulated data typed by the precise and broad algorithm; **Table S6**. Locus information from Maize B73 reference genome for simulation; **Table S7**. Four simulated situations to test SSR-typing tools.

**Additional file 2 **: **Table S8**. SSR typing results of 484 evaluated SSR loci in B73 by AMGT-TS; **Table S9**. SSR typing results of 484 evaluated SSR loci in B73 by NextGENe; **Table S10**. SSR typing results of 484 evaluated SSR loci in Jing724 by AMGT-TS; **Table S11**. SSR typing results of 484 evaluated SSR loci in Jing724 by NextGENe; **Table S12**. SSR typing results of 484 evaluated SSR loci in Jingke968 by AMGT-TS; **Table S13**. SSR typing results of 484 evaluated SSR loci in Jingke968 by NextGENe.

## Data Availability

All scripts and data used in this study could be found at https://amgt-ts.plantdna.cn/data/ and https://github.com/plantdna/amgt-ts.

## Abbreviations

PCR: Polymerase chain reaction;
BMA: Broad matching algorithm
SSR: Simple sequence repeat
STR: Short tandem repeat
SNP: Single nucleotide polymorphisms
AMGT-TS: Accurate microsatellite genotyping tool based on targeted sequencing
PIC: Polymorphism information content
CIGAR: Concise idiosyncratic gapped alignment report
