# Supplementary material for: An integrated strategy for target SSR genotyping with toleration of nucleotide variations in the SSRs and flanking regions
Source: BMC Bioinformatics. 2021 Sep 8;22:429. doi: 10.1186/s12859-021-04351-w (PMC8424866; doi:10.1186/s12859-021-04351-w)
Supplement: Supplementary file 1 — Additional file 1 : Figure S1. Artificial read composition design; Figure S2. Comparison of targeted sequencing results and resequencing results (8 samples/21 loci). This figure shows a comparison of targeted sequencing results analyzed by AMGT-TS and resequencing results (8 samples/21 loci). The abscissa is the name of each sample. The orange ordinate represents the number of loci that were compared. The green ordinate represents the same number of compared loci. Loci with missing or incomplete data were not compared. In the figure above, refer to Table S1 for the corresponding resequencing data. For the corresponding data of targeted sequencing results analyzed by AMGT-TS, please refer to Table S2. For the loci information, please refer to Table S5; Figure S3. The situation with SNP in the SSR and flanking regions; Figure S4. Comparison of SSR genotyping results between AMGT-TS and NextGENe; Figure S5. SSR typing results of two representative loci by AMGT-TS; Table S1. Genotyping information for three example loci; Table S2. This table shows the results of genotyping of 50 loci from Figure 3; Table S3. Data of Figure S2 - Resequencing data of 8 samples; Table S4. Data of Figure S2 - Targeted sequencing results analyzed by AMGT-TS; Table S5. Analysis results of simulated data typed by the precise and broad algorithm; Table S6. Locus information from Maize B73 reference genome for simulation; Table S7. Four simulated situations to test SSR-typing tools. [file 12859_2021_4351_MOESM1_ESM.docx]

**Supporting Information for:**

**An integrated strategy for target SSR genotyping with toleration of nucleotide variations in the SSRs and flanking regions**

Yongxue Huo ^a^, Yikun Zhao ^a^, Liwen Xu ^a^, Hongmei Yi ^a^, Yunlong Zhang ^a^, Xianqing Jia ^a^, Han Zhao ^b^, Jiuran Zhao ^a,*^ and Fengge Wang ^a,*^

^*^ Correspondence: wangfengge@maizedna.org (F.W.); maizezhao@126.com (J.Z.)

**Additional File 1**

This File includes:

Figure S1 to S5

Table S1 to S7


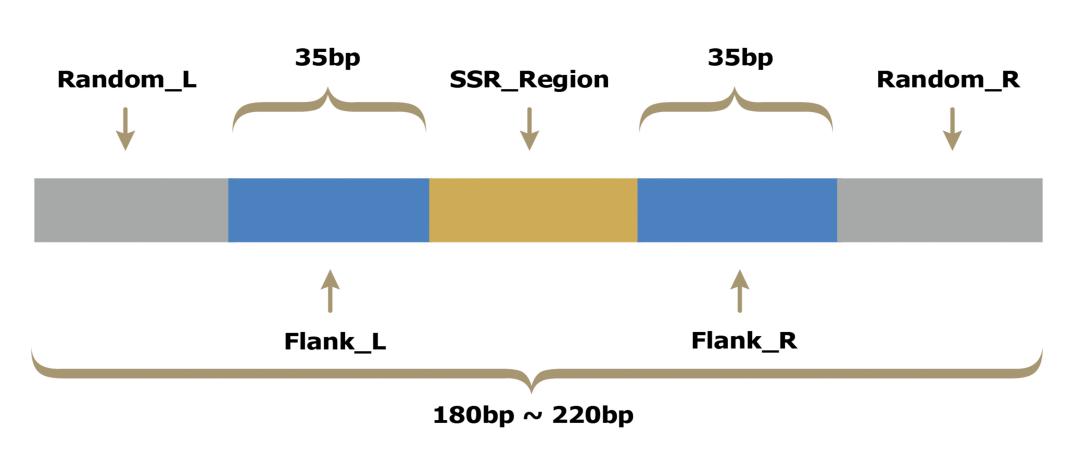


**Figure S1. Artificial read composition design.**


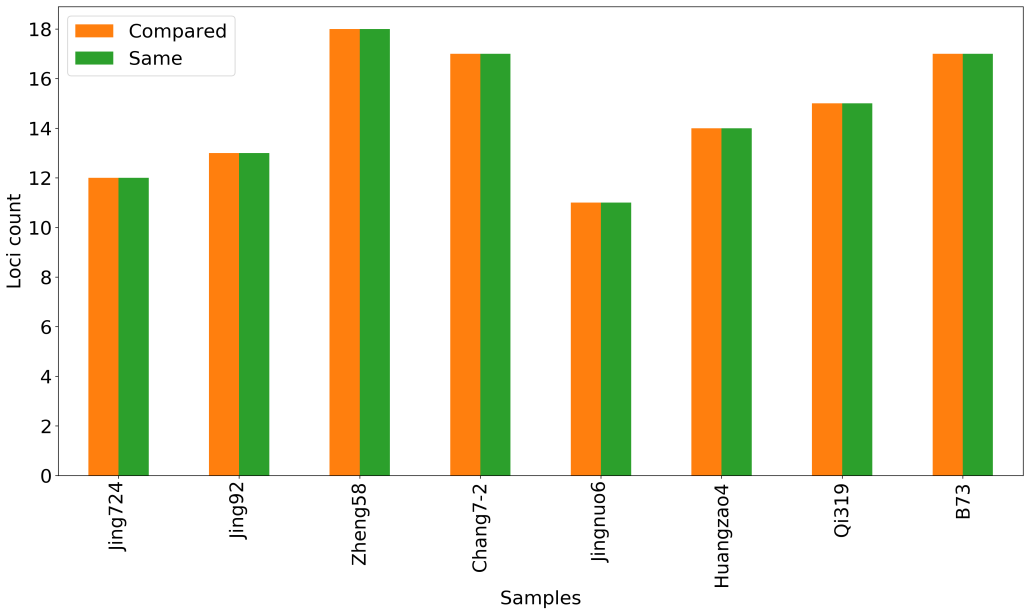


**Figure S2. Comparison of targeted sequencing results and resequencing results (8 samples / 21 loci).** This figure shows a comparison of targeted sequencing results analyzed by AMGT-TS and resequencing results (8 samples/21 loci). The abscissa is the name of each sample. The orange ordinate represents the number of loci that were compared. The green ordinate represents the same number of compared loci. Loci with missing or incomplete data were not compared. In the figure above, refer to Table S1 for the corresponding resequencing data. For the corresponding data of targeted sequencing results analyzed by AMGT-TS, please refer to Table S2. For the loci information, please refer to Table S5.


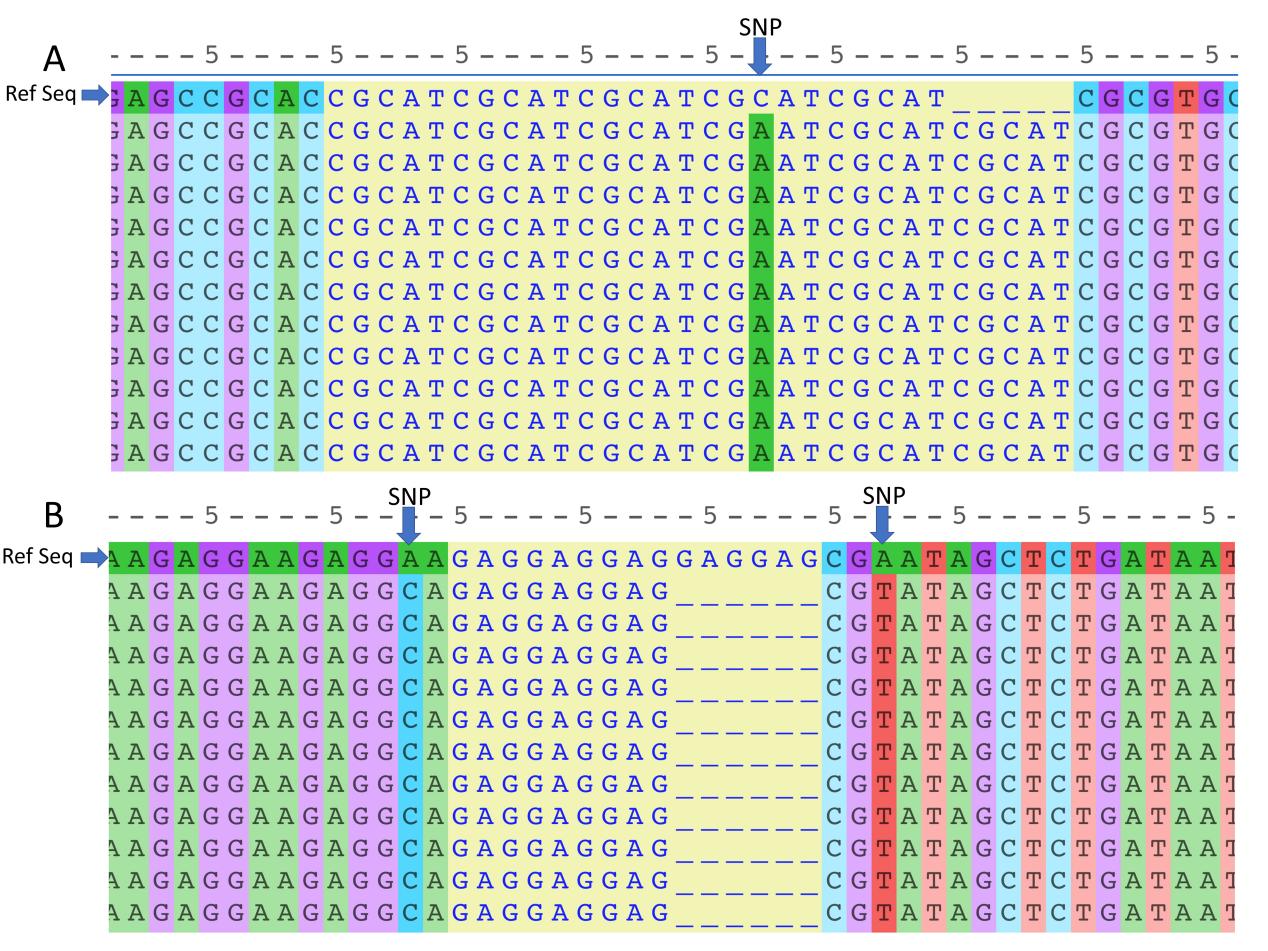


**Figure S3. The situation with SNP in the SSR and flanking regions.**


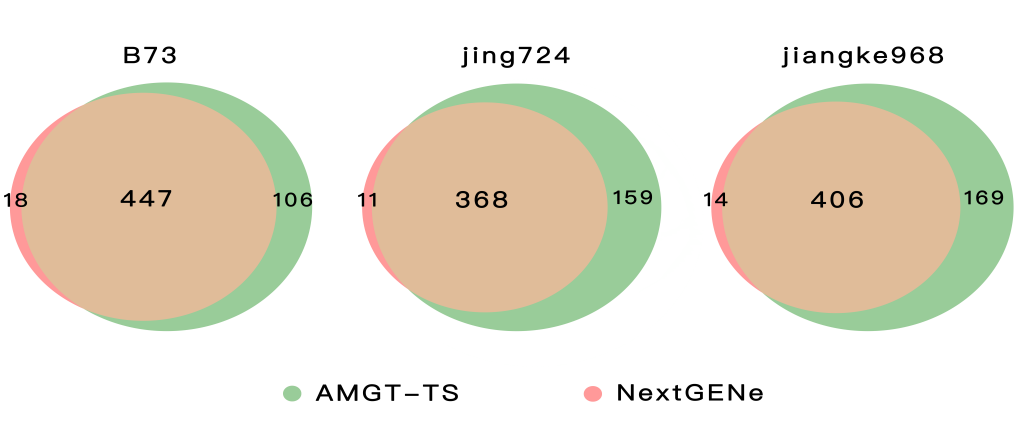


**Figure S4. Comparison of SSR genotyping results between AMGT-TS and NextGENe.**


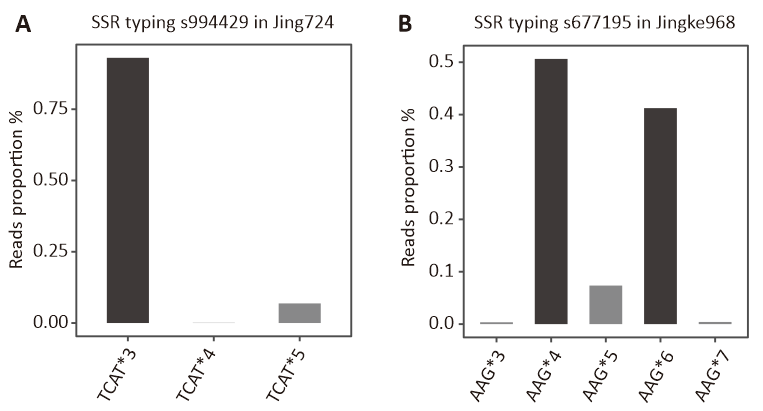


**Figure S5. SSR typing results of two representative loci by AMGT-TS.**

(A) SSR typing results of s994429 locus in Jing724 using AMGT-TS, which is a selfing variety. As expected, only one main peak (TCAT*3) was detected. (B) SSR typing results of s677195 locus in Jingke968 using AMGT-TS, which is a hybrid from two different maize varieties. Two peaks were detected, AAG*4 and AAG*6. Other genotypes with grey bars were considered to be caused by amplification stutter or sequencing error.

**Table S1. Genotyping information for three example loci.**

| Locus | Motif | SSR length | Reads number | Total reads number | Proportion |
| --- | --- | --- | --- | --- | --- |
| s17883 | ATA | 12 | 3590 | 3654 | 0.982^1^ |
|  |  | 15 | 10 | 3654 | 0.003 |
|  |  | 6 | 7 | 3654 | 0.002 |
|  |  | 9 | 47 | 3654 | 0.013 |
| s691405 | AGG | 0 | 3 | 4058 | 0.001 |
|  |  | 12 | 3995 | 4058 | 0.984^2^ |
|  |  | 15 | 7 | 4058 | 0.002 |
|  |  | 6 | 13 | 4058 | 0.003 |
|  |  | 9 | 40 | 4058 | 0.010 |
| s838417 | CTC | 12 | 9 | 1165 | 0.008 |
|  |  | 15 | 1150 | 1165 | 0.987^3^ |
|  |  | 18 | 2 | 1165 | 0.002 |
|  |  | 9 | 4 | 1165 | 0.003 |

^1^ From the “Proportion” column value (98.2%), we can get the repeat length of locus s17883 is 12. ^2^ From the “Proportion” column value (98.4%), we can get the repeat length of locus s691405 is 12. ^3^ From the “Proportion” column value (98.7%), we can get the repeat length of locus s838417 is 15.

**Table S2. This table shows the results of genotyping of 50 loci from Figure 3.**

|  | **Jingke968** | **Jing724** | **Jing92** |
| --- | --- | --- | --- |
| **s1131975** | [6, 6] | [6, 6] | [6, 6] |
| **s1181349** | [20, 8] | [20, 20] | [8, 8] |
| **s1188004** | [15, 15] | [15, 15] | [15, 15] |
| **s172002** | [6, 6] | [6, 6] | [6, 6] |
| **s205352** | [12, 12] | [12, 12] | [12, 12] |
| **s207831** | [12, 16] | [16, 16] | [12, 12] |
| **s258878** | [6, 9] | [9, 9] | [6, 6] |
| **s423645** | [20, 8] | [20, 20] | [8, 8] |
| **s458903** | [9, 9] | [9, 9] | [9, 9] |
| **s462535** | [6, 9] | [9, 9] | [6, 6] |
| **s482837** | [12, 9] | [12, 12] | [9, 9] |
| **s499955** | [9, 9] | [9, 9] | [9, 9] |
| **s657594** | [9, 9] | [9, 9] | [9, 9] |
| **s774938** | [12, 15] | [15, 15] | [12, 12] |
| **s813086** | [12, 9] | [12, 12] | [9, 9] |
| **s838195** | [9, 9] | [9, 9] | [9, 9] |
| **s948307** | [12, 12] | [12, 12] | [12, 12] |
| **s991015** | [20, 20] | [20, 20] | [20, 20] |
| **s997499** | [15, 20] | [20, 20] | [15, 15] |
| **s997901** | [15, 25] | [15, 15] | [25, 25] |
| **s1098345** | [10, 25] | [25, 25] | [10, 10] |
| **s126231** | [10, 20] | [10, 10] | [20, 20] |
| **s15146** | [8, 8] | [8, 8] | [8, 8] |
| **s156675** | [10, 15] | [10, 10] | [15, 15] |
| **s172843** | [15, 20] | [20, 20] | [15, 15] |
| **s176799** | [20, 20] | [20, 20] | [20, 20] |
| **s257671** | [9, 9] | [9, 9] | [9, 9] |
| **s307826** | [12, 9] | [9, 9] | [12, 12] |
| **s425466** | [12, 12] | [12, 8] | [12, 12] |
| **s459130** | [9, 9] | [9, 9] | [9, 9] |
| **s490622** | [12, 12] | [12, 12] | [12, 12] |
| **s508769** | [12, 9] | [12, 12] | [9, 9] |
| **s558408** | [12, 16] | [16, 16] | [12, 12] |
| **s559159** | [12, 12] | [12, 12] | [12, 12] |
| **s626333** | [12, 6] | [12, 12] | [6, 6] |
| **s6496** | [10, 10] | [10, 10] | [10, 10] |
| **s671420** | [20, 20] | [20, 20] | [20, 20] |
| **s796946** | [15, 20] | [20, 20] | [15, 15] |
| **s798171** | [20, 20] | [20, 20] | [20, 20] |
| **s798464** | [20, 25] | [20, 20] | [25, 25] |
| **s890539** | [12, 16] | [16, 16] | [12, 12] |
| **s895099** | [15, 20] | [20, 20] | [15, 15] |
| **s898387** | [9, 9] | [9, 9] | [9, 9] |
| **s922742** | [6, 6] | [6, 6] | [6, 6] |
| **s93347** | [12, 12] | [12, 12] | [12, 12] |
| **s945463** | [6, 9] | [6, 6] | [9, 9] |
| **s992385** | [16, 20] | [16, 16] | [20, 20] |
| **s996971** | [15, 20] | [15, 15] | [20, 20] |
| **s997242** | [25, 25] | [25, 25] | [25, 25] |
| **s997741** | [10, 15] | [10, 10] | [15, 15] |

**Table S3. Data of Figure S2 - Resequencing data of 8 samples.**

| Locus | Jing724 (CX59) | Jing92 (CX60) | Zheng58 (CX38) | Chang7-2 (CX310) | Jingnuo6 (CX62) | Huangzao4 (CX14) | Qi319 (CX37) | B73 (CX67) |
| --- | --- | --- | --- | --- | --- | --- | --- | --- |
| s1081917 | - | t6 | t7 | t6 | t8 | t6 | - | - |
| s1093447 | t6 | t4 | t6 | t5 | t3 | t5 | - | - |
| s1183267 | - | - | t5 | t3 | t5 | t3 | t3 | t5 |
| s149275 | t5 | - | t6 | - | - | - | t4 | - |
| s17883 | t7 | t6 | t4 | t6 | - | t4 | t7 | t4 |
| s26593 | t8 | t8 | t8 | t8 | - | t8 | t6 | t7 |
| s293944 | - | - | t3 | t2 | t2 | - | t2 | t4 |
| s4121 | - | - | - | - | - | - | - | t6 |
| s426991 | t5 | t4 | t4 | t4 | t5 | t4 | t4 | t6 |
| s433896 | t2 | t4 | t3 | t4 | t4 | t4 | t3 | t3 |
| s457102 | - | t7 | t7 | t7 | t6 | t6 | t4 | t7 |
| s470006 | t6 | t7 | t3 | t7 | - | t7 | t7 | t6 |
| s48611 | t6 | - | t7 | t6 | - | t4 | - | t5 |
| s600659 | t9 | t4 | t9 | t4 | - | t4 | - | t10 |
| s642349 | t7 | t9 | t5 | t9 | t6 | - | t9 | t6 |
| s691405 | - | t2 | - | t2 | - | - | t5 | t4 |
| s746628 | - | - | - | t6 | t6 | - | t6 | t6 |
| s795376 | t4 | - | t7 | t3 | - | - | t3 | t7 |
| s827114 | - | - | t3 | - | t5 | t6 | t3 | t5 |
| s838417 | - | t4 | t6 | t4 | t5 | t4 | - | t5 |
| s893539 | t8 | t8 | t11 | - | - | t11 | t3 | - |

**Table S4. Data of Figure S2 - Targeted sequencing results analyzed by AMGT-TS.**

| Locus | Motif | Jing724 | Jing92 | Zheng58 | Chang7-2 | Jingnuo6 | Huangzao4 | Qi319 | B73 |
| --- | --- | --- | --- | --- | --- | --- | --- | --- | --- |
| s1081917 | TGG | 21,21 | 18,18 | 21,21 | 18,18 | 24,24 | 18,18 | 24,24 | 27,27 |
| s1093447 | GAGC | 24,24 | 16,16 | 24,24 | 20,20 | 12,12 | 20,20 | 24,24 | 24,24 |
| s1183267 | CCAT | 24,24 | 20,20 | 20,20 | 12,12 | 20,20 | 12,12 | 12,12 | 20,20 |
| s149275 | CTC | 15,15 | 9,9 | 18,18 | 18,18 | 18,18 | 9,9 | 12,12 | 18,18 |
| s17883 | ATA | 21,21 | 18,18 | 12,12 | 18,18 | 12,12 | 12,12 | 21,21 | 12,12 |
| s26593 | CCT | 24,24 | 24,24 | 24,24 | 24,24 | 9,9 | 24,24 | 18,18 | 21,21 |
| s293944 | TGCG | 12,12 | 8,8 | 12,12 | 8,8 | 8,8 | 8,8 | 0,8 | 16,16 |
| s4121 | AGAGA | 20,20 | 35,35 | 15,15 | 35,35 | 35,35 | 35,35 | 20,20 | 30,30 |
| s426991 | GATA | 20,20 | 16,16 | 16,16 | 16,16 | 20,20 | 16,16 | 16,16 | 24,24 |
| s433896 | TGTTG | 10,10 | 20,20 | 15,15 | 20,20 | 20,20 | 20,20 | 15,15 | 15,15 |
| s457102 | ATA | 12,12 | 21,21 | 21,21 | 21,21 | 18,18 | 18,18 | 12,12 | 21,21 |
| s470006 | CAG | 18,18 | 21,21 | 9,9 | 21,21 | 18,18 | 21,21 | 21,21 | 18,18 |
| s48611 | GAG | 18,18 | 12,12 | 21,21 | 18,18 | 15,15 | 12,12 | 21,21 | 15,15 |
| s600659 | CCT | 27,27 | 12,12 | 27,27 | 12,12 | 12,12 | 12,12 | 27,27 | 30,30 |
| s642349 | TCA | 21,21 | 27,27 | 15,15 | 27,27 | 18,18 | 27,27 | 27,27 | 18,18 |
| s691405 | AGG | 15,15 | 6,6 | 12,6 | 6,6 | 6,6 | 6,6 | 15,15 | 12,12 |
| s746628 | GGA | 18,18 | 18,18 | 24,24 | 18,18 | 18,18 | 18,18 | 18,18 | 18,18 |
| s795376 | TTCT | 16,16 | 12,12 | 28,28 | 12,12 | 24,24 | 24,24 | 12,12 | 28,28 |
| s827114 | CCA | 9,9 | 18,18 | 9,9 | 15,15 | 15,15 | 18,18 | 9,9 | 15,15 |
| s838417 | CTC | 9,9 | 12,12 | 18,18 | 12,12 | 15,15 | 12,12 | 18,18 | 15,15 |
| s893539 | TTCT | 32,32 | 32,32 | 44,44 | 32,32 | 44,44 | 44,44 | 12,12 | 32,32 |

**Table S5. Analysis results of simulated data typed by the precise and broad algorithm.**

| Locus | Motif | Total reads number | Precise algorithm | | | Broad algorithm | | |
| --- | --- | --- | --- | --- | --- | --- | --- | --- |
|  |  |  | SSR length | Reads number | Proportion | SSR length | Reads number | Proportion |
| s423645 | AGCT | 2000 | 20 | 1000 | 0.5 | 20 | 1000 | 0.5 |
|  |  | 2000 | 24 | 1000 | 0.5 | 24 | 1000 | 0.5 |
| s499955 | GAG | 2000 | 9 | 2000 | 1 | 9 | 2000 | 1 |
| s996971 | CGCAT | 4000 | 15 | 2000 | 0.5 | 20 | 2000 | 0.5 |
|  |  | 4000 | 20 | 2000 | 0.5 | 30 | 2000 | 0.5 |

Regarding the column headings, Locus is the locus name; Motif is a sequence of repeating units of the SSR; SSR length is the total length of SSR region, that is, the genotyping length; Reads number is the corresponding read number of the current SSR genotyping length; Total reads number is the total number of reads at this locus; Proportion represents the proportion of the corresponding read number of the current SSR genotyping length relative to the total number of reads.

**Table S6. Locus information from Maize B73 reference genome for simulation.**

| ID | Name | Motif | Source | Chrome | Position range | PIC |
| --- | --- | --- | --- | --- | --- | --- |
| Locus1 | s258878 | GCT | Maize B73V3 | Chr10 | 1726174~1726182 | 0.7213 |
| Locus2 | s423645 | AGCT | Maize B73V3 | Chr2 | 197798028~197798047 | 0.4958 |
| Locus3 | s566749 | GCTTT | Maize B73V3 | Chr3 | 7281270~7281289 | 0.5315 |

**Table S7. Four simulated situations to test SSR-typing tools.**

| ID | Category Description | Loci count | Length(bp) | Total reads | Repeats of motifs | | | Reads grouped by locus | | |
| --- | --- | --- | --- | --- | --- | --- | --- | --- | --- | --- |
|  |  |  |  |  | Locus1 | Locus2 | Locus3 | Locus1 | Locus2 | Locus3 |
| A | No variant in SSR or flanking region | 3 | 180~220 | 30000 | 5 | 3 | 3 | 10k | 10k | 10k |
| B | One SNP in SSR region | 3 | 180~220 | 30000 | 5 | 3 | 3 | 10k | 10k | 10k |
| C | One SNP in flanking region | 3 | 180~220 | 30000 | 5 | 3 | 3 | 10k | 10k | 10k |
| D | 2-bp deletion in flanking region | 3 | 180~220 | 30000 | 5 | 3 | 3 | 10k | 10k | 10k |
